# Clinical characteristics, specific resistance patterns, and molecular mechanisms of carbapenem-resistant *Morganella morganii* isolates

**DOI:** 10.3389/fcimb.2025.1672736

**Published:** 2025-09-05

**Authors:** Xiangkuo Zheng, Weiliang Zeng, Yan Liu, Luozhu Feng, Jiayin Zheng, Tieli Zhou, Changrui Qian, Cui Zhou

**Affiliations:** ^1^ School of Medicine, Huzhou University; Key Laboratory of Vector Biology and Pathogen Control of Zhejiang Province, Huzhou, Zhejiang, China; ^2^ Department of Clinical Laboratory, the First Affiliated Hospital of Wenzhou Medical University, Key Laboratory of Clinical Laboratory Diagnosis and Translational Research of Zhejiang Province, Wenzhou, Zhejiang, China; ^3^ Department of Clinical Laboratory, the First Affiliated Hospital of Ningbo University, Ningbo, Zhejiang, China

**Keywords:** *Morganella morganii*, carbapenem-resistant, imipenem, penicillin-binding protein, penicillin-binding protein activator

## Abstract

**Objectives:**

The emergence and spread of carbapenem-resistant *Morganella morganii* (*M. morganii*) pose a serious global challenge. This study aimed to investigate the clinical characteristics, resistance patterns, and molecular mechanisms of carbapenem-resistant *M. morganii*.

**Methods:**

A total of 170 *M. morganii* clinical isolates were collected from the First Affiliated Hospital of Wenzhou Medical University (Wenzhou, China) between January 2016 and December 2017. Carbapenem MICs were determined by antimicrobial susceptibility testing. Carbapenem resistance determinants, including carbapenemase genes (*bla*_KPC-2_, *bla*_VIM_, *bla*_IMP_, *bla*_NDM_, and *bla*_OXA-48_) and extended-spectrum β-lactamase (ESBL) genes (*bla*_TEM_, *bla*_CTX-M-1_, and *bla*_SHV_), were analyzed by polymerase chain reaction (PCR). PCR and sequencing assays were performed to detect penicillin-binding protein (PBP) mutations. Efflux pump activity was also assessed in carbapenem-resistant isolates. Quantitative real-time PCR (qRT-PCR) was used to determine the relative mRNA expression levels of outer membrane porin-encoding gene *ompC* and PBP activator-encoding genes *lpoA* and *lpoB*.

**Results:**

Twenty-six imipenem-resistant and 108 imipenem-intermediate *M. morganii* isolates were identified, accounting for 15.29% and 63.53% of cases, respectively. No isolates were resistant to meropenem or ertapenem. Among the 26 carbapenem-resistant isolates, the prevalence of ESBL genes *bla*_TEM_ and *bla*_CTX-M-1_ was 30.77% and 11.54%, respectively, while carbapenemase genes were not detected. Resistant isolates carried more specific PBP mutations than carbapenem-susceptible and carbapenem-intermediate isolates. Efflux pump phenotypes were associated with reduced imipenem susceptibility in 13 carbapenem-resistant isolates. qRT-PCR revealed no significant differences in *ompC* expression among the resistant, intermediate, and susceptible groups; however, significant differences were observed in *lpoA* and *lpoB* expression. Isolates in the imipenem-resistant group carried more PBP mutations.

**Conclusion:**

*M. morganii* isolates were commonly non-susceptible to imipenem but remained susceptible to meropenem and ertapenem. Low expression of PBP activator genes (*lpoA* and *lpoB*), along with the presence of specific PBP mutations, appeared to be the primary mechanisms of resistance. In addition, efflux pump overexpression may contribute to imipenem resistance in *M. morganii*.

## 1 Introduction

*Morganella morganii* (*M. morganii*), a facultative anaerobic *Gram*-negative bacterium, is the only *species* in the genus *Morganella* of the *Enterobacteriaceae* family. It is divided into two subspecies: *Morganella* subspecies and siboni subspecies (Liu et al., 2016; Chen et al., 2024; Liu et al., 2025). This bacterium is widely found in the natural environment and in the intestines of humans, mammals, and reptiles. *M. morganii* is an important opportunistic pathogen in clinical settings, often causing urinary tract infections after catheterization and postoperative wound infections. It has also been reported to cause sepsis, meningitis, pneumonia, arthritis, and other nosocomial infections (Erlanger et al., 2019; Bandy, 2020; Zaric et al., 2021; Laupland et al., 2022). Due to increasing bacterial resistance to third-generation cephalosporins, aminoglycosides, and fluoroquinolones, carbapenems have gradually become the last option for treating multidrug-resistant *M. morganii* infections, which pose life-threatening health risks (Guo et al., 2019; Bandy, 2020; Park et al., 2020). However, with the wide application of carbapenems in clinical practice, the global spread of carbapenem-resistant pathogens has brought great challenges to public health worldwide (Bonnin et al., 2024; Yao et al., 2025).

Several studies have reported that carbapenem resistance in *M. morganii* is associated not only with harboring carbapenemases, including KPC, NDM, IMP, VIM, and OXA, and extended-spectrum β-lactamases (ESBLs), but also with the overexpression of efflux pumps (Guo et al., 2019; Shrestha et al., 2020; Bonnin et al., 2024). In addition, the outer membrane structure of this pathogen can effectively prevent harmful substances from entering bacterial cells. However, the loss or decreased expression of outer membrane porins in *M. morganii* contributes to the multidrug-resistant (MDR) phenotype (Nordmann et al., 2012; Logan and Weinstein, 2017).

In Gram-positive bacteria, β-lactamases and permeability barriers play a limited role in drug resistance, so penicillin-binding protein (PBP)-related mechanisms have been studied extensively in this group. By contrast, the role of PBPs in drug resistance among Gram-negative bacteria has often been ignored. In recent years, increasing attention has been paid to PBP-related resistance in Gram-negative bacteria, with studies focusing on PBPs as antibiotic targets from different perspectives, which has important implications for the treatment of infectious diseases (Aissa et al., 2016; Aitken et al., 2024; Tellapragada et al., 2024; Fabrizio and Tascini, 2025). Carbapenems inhibit cell wall synthesis by binding to bacterial PBPs, including the high-molecular-weight enzymes PBP1a, PBP1b, PBP2, and PBP3 (Shalaby et al., 2020; Bertonha et al., 2023; Hu and Wang, 2024). In *Enterobacteriaceae*, different carbapenems have varying affinities for PBPs. Imipenem has higher affinity for PBP1a and PBP1b, binding two to four times more strongly than other carbapenems (Aissa et al., 2016; Fabrizio and Tascini, 2025). Substitution of amino acids in PBPs or the acquisition of new PBPs can lead to bacterial resistance to carbapenems. PBP modification often contributes to resistance to β-lactam antibiotics in both Gram-positive and Gram-negative bacteria; however, PBP modification alone rarely results in high levels of resistance to carbapenems (Cherkaoui et al., 2017; Malik et al., 2020). With the increasing prevalence of carbapenem-resistant *M. morganii* worldwide, it is of great clinical importance to explore their specific resistance patterns and analyze molecular epidemiology to prevent and control the occurrence and transmission of resistance, as well as to guide antimicrobial therapy (Guo et al., 2019; Xiang et al., 2021).

In this study, 170 *M. morganii* strains clinically isolated at our hospital in southeastern China between January 2016 and December 2017 were retrospectively analyzed. We characterized the specific resistance patterns and molecular mechanisms of 26 imipenem-resistant isolates that remained susceptible to meropenem and ertapenem.

## 2 Materials and methods

### 2.1 Bacterial strains

A total of 170 *M. morganii* clinical isolates were collected from the First Affiliated Hospital of Wenzhou Medical University (Wenzhou, China) between January 2016 and December 2017. All isolates were identified as *M. morganii* using the VITEK^®^2 mass spectrometry (MS) system (bioMérieux, France). After collection, isolates were stored at −80 °C in Luria–Bertani (LB) broth with 30% sterilized glycerol. Relevant clinical data, including date of isolation, patient age, sex, sample type, and ward, were retrieved from medical records.

### 2.2 Minimum inhibitory concentration determination

According to the latest guidelines version CLSI M100–2025 recommendedby the Clinical and Laboratory Standards Institute (CLSI, 2025), we measured the MICs by agar dilution method, including imipenem, meropenem, and ertapenem in this study. Briefly, after overnight culture of single colonies, the suspension was adjusted to 0.5 McFarland (approximately 1.5 × 10^8^ CFU/mL) in sterilized NaCl. Following a 10-fold dilution, suspensions were evenly spread on medicated Mueller–Hinton agar plates and incubated at 37 °C for 16–18 h. Results were recorded after incubation. Meropenem and ertapenem were dissolved in sterile water, while imipenem was dissolved in sterile phosphate-buffered saline (PBS, 0.01 mol/L, pH 7.2), and tested over a concentration range of 0.0125–16 µg/mL. *Escherichia coli* (*E. coli*) strain ATCC25922 was used as the quality control strain. MIC values were determined in at least three independent experiments. Twenty-six imipenem-resistant *M. morganii* strains that remained susceptible to meropenem and ertapenem were selected for further analysis of resistance mechanisms.

### 2.3 Determinations of carbapenemases and extended-spectrum β-lactamase

Polymerase chain reaction (PCR) and sequencing assays were performed to detect carbapenem resistance determinants, including carbapenemase genes (*bla*_KPC-2_, *bla*_VIM_, *bla*_IMP_, *bla*_NDM_, and *bla*_OXA-48_), and ESBL genes (*bla*_TEM_, *bla*_CTX-M-1_, and *bla*_SHV_), in the 26 imipenem-resistant *M. morganii* isolates. Genomic DNA was extracted using a commercial Genomic DNA Extraction Kit (Qiagen). **Supplementary Table S1** showed the primers used to amplify DNA templates in this study. Electrophoresis was performed using 1% agarose gels. Subsequently, these resulted positive amplifications were commissioned to Shanghai BGI Technology Co. (China) for sequencing. The nucleotide sequences were compared by searching the GenBank using BLAST (http://blast.ncbi.nlm.nih.gov/Blast.cgi).

### 2.4 Efflux pump activity on imipenem-resistant *M. morganii*

The methodology for the efflux pump inhibition test has been described in detail in a previous publication (Zheng et al., 2022). Efflux pump inhibitors carbonyl cyanide m-chlorophenylhydrazone (CCCP), N-methylpyrrolidone (NMP), and Phe-Arg-β-naphthylamide (pAβN) were used. First, the agar dilution method was applied to determine the appropriate concentrations of CCCP, pAβN, and NMP that inhibited efflux activity without affecting bacterial growth. The final concentrations were 2 μg/mL, 50 μg/mL, and 128 μg/mL, respectively. MICs of imipenem against 26 imipenem-resistant *M. morganii* strains were then determined on Mueller–Hinton agar plates with or without the inhibitors. A ≥4-fold reduction in imipenem MIC in the presence of an inhibitor was considered evidence of efflux pump activity (Zheng et al., 2022).

### 2.5 PBP mutation analysis

Mutations in PBP1a, PBP1c, and PBP2 of all 26 imipenem-resistant *M. morganii* and *M. morganii* ATCC25830 strains were analyzed by PCR, while 15 imipenem-intermediate and 15 imipenem-susceptible *M. morganii* strains were analyzed in parallel. Genomic DNA was extracted using a commercial Genomic DNA Extraction Kit (Qiagen). The primers used for amplification are listed in **Supplementary Table S1**. Electrophoresis was performed on 1% agarose gels. Positive amplicons were sequenced by Shanghai BGI Technology Co. (China). Nucleotide sequences were compared with reference sequences in GenBank using BLAST (http://blast.ncbi.nlm.nih.gov/Blast.cgi).

### 2.6 Quantitative real-time polymerase chain reaction

All 26 imipenem-resistant *M. morganii and M. morganii* ATCC25830 strains were cultured to an OD_600_ of 0.5~0.6 (the logarithmic growth phase) in fresh Luria–Bertani (LB) broth at 37°C/180 rpm. The total RNA of 26 imipenem-resistant *M. morganii and M. morganii* ATCC25830 strains were extracted using the Bacterial RNA Miniprep Kit (Biomiga, Shanghai, China). In addition, 15 imipenem-intermediate and 15 imipenem-susceptible *M. morganii* strains were analyzed in parallel. A total of 100 ng RNA was reverse-transcribed into first-strand cDNA using the PrimeScript™ RT reagent Kit (Perfect Real Time) (Takara, Japan). The primers used in this study are listed in **Supplementary Table S1**. The *16S rRNA* gene was used as an endogenous control. Expression levels of the outer membrane porin-encoding gene *ompC* and the PBP activator-encoding genes *lpoA* and *lpoB* were determined by qRT-PCR using TB Green Premix Ex Taq II (Tli RNase H Plus) (2×) (Takara, Japan). Gene transcript levels were compared among resistant, intermediate, and susceptible groups using Student’s *t*-test. Expression levels of each gene in *M. morganii* ATCC25830 strains were normalized to a value of 1.0. The 2^-ΔΔCt^ method was applied for data analysis.

### 2.7 Statistical analysis

All experimental procedures were performed in triplicate with independent biological replicates. Quantitative data are presented as mean ± standard deviation (SD). Intergroup comparisons were analyzed using one-way ANOVA. Statistical analyses were performed using GraphPad Prism, version 9.02 (GraphPad Software Inc., San Diego, CA, USA). Two-tailed tests were used throughout, and *P* < 0.05 was considered statistically significant.

## 3 Results

### 3.1 Antimicrobial susceptibility profiles and clinical characteristics

The results of antimicrobial susceptibility testing revealed that 26 imipenem-resistant and 108 imipenem-intermediate *M. morganii* isolates were identified among the 170 isolates tested, accounting for 15.29% and 63.53%, respectively. However, all 170 *M. morganii* isolates were susceptible to meropenem and ertapenem (**Table 1**). In addition, 26 carbapenem-resistant *M. morganii* isolates were mainly from wound samples (38.46%, 10/26), followed by urine (19.23%, 5/26) and pus (15.38%, 4/26). There were more isolates from males than females (76.92% (20/26) *vs* 23.08% (6/26), respectively). Isolates were collected from patients aged 21 to 91 years (mean age, 68.27 years). The majority of the isolates were from patients in the wound center, urology, and endocrinology departments.

**Table 1:** Antimicrobial susceptibility profiles and clinical characteristics of 26 carbapenem-resistant *M. morganella* isolates.

Isolates	MIC (μg/mL)			Isolation date	Age	Gender ^d^	Sample	Ward
	IPM ^a^	MEM ^b^	ETP ^c^					
SL-509	8	0.125	0.015	2016/12/14	74	F	Wound	Endocrinology
SL-515	8	0.125	0.03	2016/12/23	91	M	Urine	Urology
SL-531	8	0.125	0.015	2017/1/10	21	M	Pus	Transplantation
SL-543	8	0.5	0.06	2017/1/31	83	M	Drainage	ICU
SL-545	8	0.125	0.03	2017/2/1	48	M	Sputum	Gastroenterology
SL-546	8	0.125	0.03	2017/2/6	60	M	Urine	Urology
SL-551	4	0.125	0.03	2017/2/15	70	M	Sputum	Neurosurgery
SL-570	8	0.25	0.03	2017/3/9	84	M	Wound	Wound Center
SL-572	4	0.125	0.03	2017/3/10	67	M	Wound	Endocrinology
SL-582	8	0.125	0.03	2017/3/23	82	F	Sputum	ICU
SL-596	4	0.125	0.015	2017/4/21	81	M	Wound	Wound Center
SL-603	8	0.125	0.015	2017/4/22	86	M	Drainage	ICU
SL-611	8	0.125	0.015	2017/5/2	79	M	Wound	Wound Center
SL-637	8	0.25	0.03	2017/6/19	75	M	Wound	Endocrinology
SL-640	8	0.25	0.06	2017/6/20	61	M	Blood	Gastroenterology
SL-650	4	0.125	0.015	2017/7/10	69	M	Wound	Endocrinology
SL-655	8	0.125	0.03	2017/7/20	49	M	Wound	Wound Center
SL-661	8	0.125	0.015	2017/8/1	64	M	Pus	Orthopedics
SL-663	8	0.125	0.03	2017/8/3	42	M	Pus	Anorectal Surgery
SL-666	4	0.125	0.03	2017/8/11	83	M	Wound	Wound Center
SL-706	8	0.25	0.06	2017/10/10	71	F	Urine	Urology
SL-716	4	0.125	0.015	2017/10/18	74	F	Urine	Vascular cardiology
SL-719	4	0.125	0.015	2017/10/19	55	M	Wound	Otorhinolaryngology
SL-726	8	0.125	0.015	2017/10/26	64	M	Urine	Urology
SL-749	4	0.125	0.015	2017/11/29	74	F	Pus	Endocrinology
SL-755	4	0.25	0.03	2017/12/12	68	F	Other	Otorhinolaryngology

a imipenem.

b meropenem.

c ertapenem.

d M, Male; F, Female.

### 3.2 Carbapenemases and β-lactamase genes prevalence

**Figure 1:**
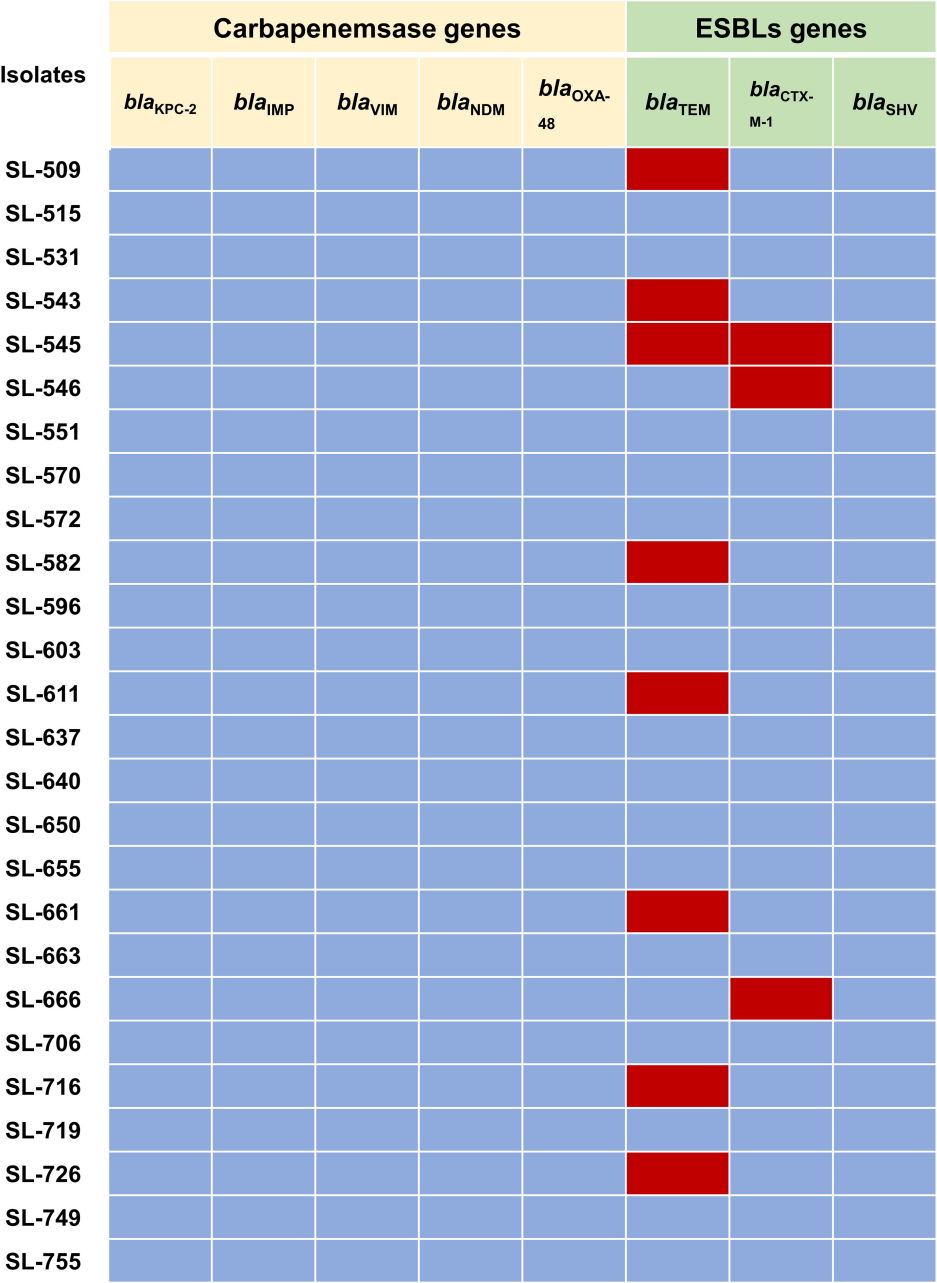
Prevalence of carbapenemases-encoding genes and ESBLs-encoding genes in 26 imipenem-resistant *M. morganii* isolates. Red colours indicate presence while blue colours represent absence.

The mechanisms of carbapenem resistance in the 26 imipenem-resistant *M. morganii* isolates were investigated by PCR. No carbapenemase-encoding genes (*bla*_KPC-2_, *bla*_NDM_, etc.) were detected. By contrast, ESBL genes were identified: *bla*_TEM_ was present in 30.77% of isolates and *bla*_CTX-M-1_ in 11.54%, while *bla*_SHV_ was not detected (**Figure 1**).

### 3.3 Phenotypic detection of the efflux pump overexpression

A series of efflux pump inhibitors was tested for their effect on imipenem susceptibility, including CCCP, pAβN, and NMP. Imipenem MICs of the 26 imipenem-resistant *M. morganii* isolates decreased by less than twofold when exposed to pAβN and NMP. However, imipenem MICs decreased by more than fourfold in the presence of 2 μg/mL CCCP 13 imipenem-resistant isolates, confirming an association between efflux pump phenotypes and reduced imipenem susceptibility in these strains (**Table 2**).

**Table 2:** Efflux pump phenotype test.

Isolates	MICs (μg/mL)							Efflux pump Phenotype ^b^
	IPM	IPM + CCCP (2 μg/mL)	Fold changes ^a^	IPM + pAβN (50 μg/mL)	Fold changes	IPM + NMP (128 μg/mL)	Fold changes	
SL-509	8	0.5	16	4	2	4	2	+
SL-515	8	2	4	4	2	4	2	
SL-531	8	2	4	4	2	4	2	+
SL-543	8	4	2	8	1	8	1	–
SL-545	8	2	4	4	2	4	2	+
SL-546	8	2	4	4	2	4	2	+
SL-551	4	2	2	4	1	2	2	-
SL-570	8	4	2	4	2	4	2	–
SL-572	4	2	2	4	1	4	1	-
SL-582	8	2	4	4	2	4	2	+
SL-596	4	4	1	4	1	4	1	-
SL-603	8	2	4	4	2	4	2	+
SL-611	8	2	4	4	2	4	2	+
SL-637	8	2	4	4	2	4	2	+
SL-640	8	4	2	4	2	4	1	-
SL-650	4	2	2	4	1	4	1	–
SL-655	8	2	4	4	2	4	1	+
SL-661	8	4	2	4	2	4	1	–
SL-663	8	2	4	4	2	2	1	+
SL-666	4	2	2	4	1	2	1	–
SL-706	8	4	2	4	2	4	1	-
SL-716	4	2	2	4	2	2	1	–
SL-719	4	1	4	4	1	4	1	+
SL-726	8	2	4	4	2	4	1	+
SL-749	4	4	1	4	1	4	1	-
SL-755	4	4	1	4	1	4	1	–

a Ratio of MIC without inhibitor to MIC with inhibitor.

b Compared with imipenem alone, the MICs value of imipenem decreased ≥4 was confirmed to have an inhibitory effect when imipenem was used in combination with efflux pump inhibitors; ^+^ indicates the strains with positive efflux pump phenotype; ^-^ indicates the strains with negative efflux pump phenotype.

### 3.4 Determination of PBP mutations

The genes *mrcA*, *pbpC*, and *mdrA* encode the three primary PBPs in *M. morganii*: PBP1a, PBP1c, and PBP2, respectively. PCR results demonstrated that imipenem-resistant *M. morganii* isolates carried more specific PBP mutations than the imipenem-susceptible and imipenem-intermediate strains, such as PBP1c-Glu76Gln, PBP1c-Asn235Asp, PBP1c-Thr475Ile, PBP1c-Val783Met, and PBP2-Gly487Ser (**Table 3**). **Supplementary Table S2** details all PBP missense mutations in the imipenem-resistant, intermediate, and susceptible isolate groups.

**Table 3:** Analysis of PBPs mutations in 26 IPM-resistant *M. morganii* isolates.

Gene	Gene length (bp)	Missense mutations^a^
PBP1a (*mrcA*)	2526	1198G>A p.Gly400Ser, 914C>G p.Thr305Ser, 276C>G p.Asp92Glu, 2496G>T p.Gln832His, 1811C>T p.Thr604Ile, 1303A>G p.Thr435Ala, 994G>A p.Asp332Asn, 469G>A p.Asp157Asn, 422T>C p.Val141Ala,
PBP1c (*pbpC*)	2361	682T>C p.Trp228Arg, 1034C>T p.Ala345Val, 1454G>A p.Arg485His, 56C>T p.Thr19Ile, 1940A>G p.Gln647Arg, 109G>C p.Val37Leu, 629G>C p.Gly210Ala, 838G>A p.Val280Ile, 2008C>A p.Leu670Met, 2214G>T p.Glu738Asp, 178C>T p.Arg60Cys, 398T>C p.Leu133Pro, 698C>A p.Pro233Gln, 913G>A p.Ala305Thr, 1001C>T p.Thr334Met, 1402G>A p.Val468Ile, 1532A>G p.Gln511Arg, 1966A>G p.Ile656Val, 2107C>T p.Arg703Cys, 2257A>C p.Lys753Gln, 1726A>G p.Ile576Val, 1697G>T p.Gly566Val, 1426A>C p.Ile476Leu, 855G>A p.Met285Ile, 608G>A p.Arg203His, 523A>G p.Ser175Gly
PBP2 (*mdrA*)	1881	1546T>C p.Phe516Leu, 1495G>A p.Ala499Thr, 1574C>T p.Thr525Ile

a Predict by PROVEAN software and compared with sequences of *M. Morganella* ATCC 25830 in GenBank.

### 3.5 Expression of gene encoding outer membrane porin

**Figure 2:**
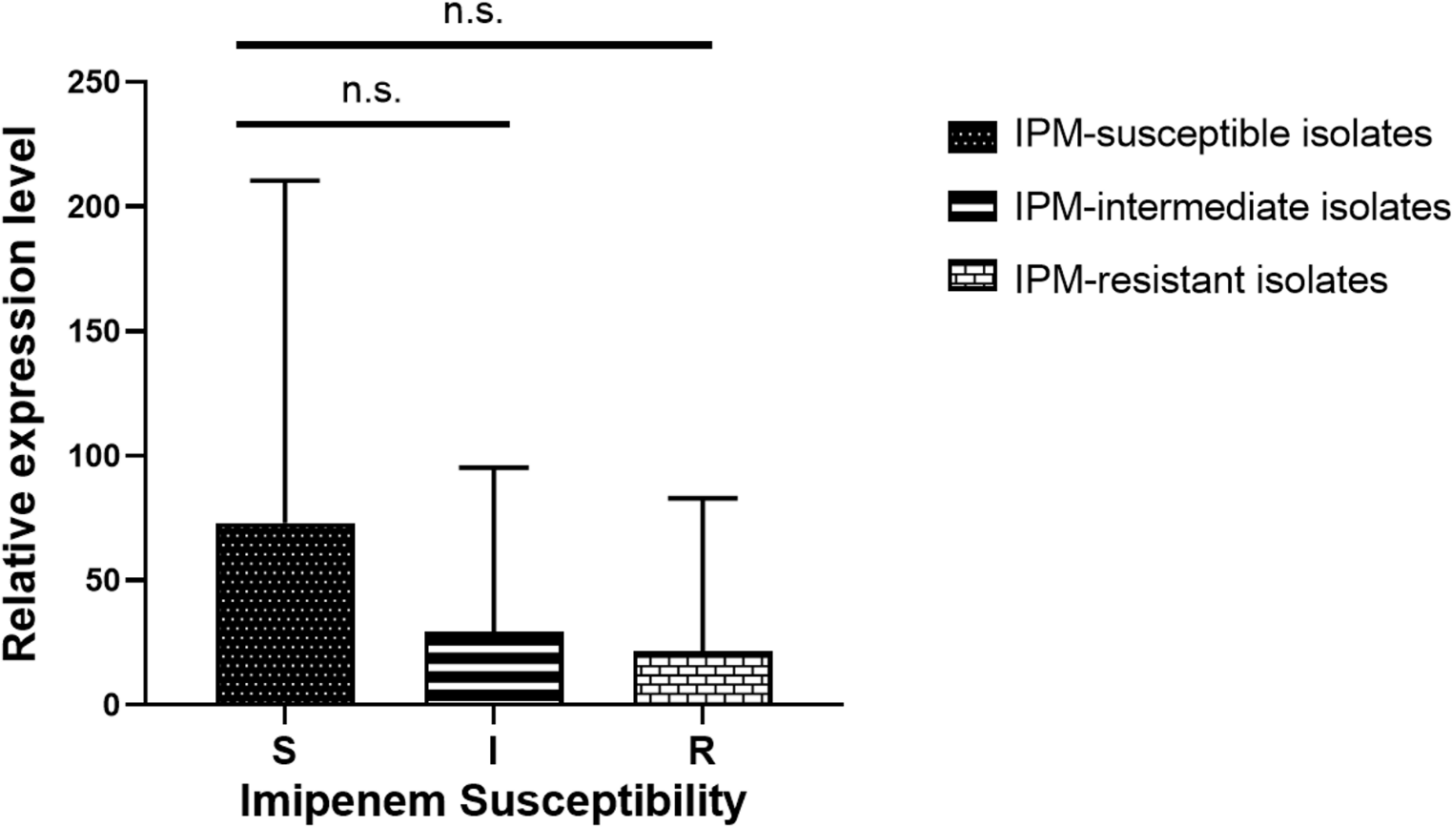
Transcript levels of outer membrane porin-encoding gene *ompC* among the 26 imipenem-resistant *M. morganii*, 15 imipenem-intermediate *M. morganii* strains, 15 imipenem-susceptible *M. morganii* groups. n.s., *P* >0.05.

A study was conducted to examine the relationship between imipenem resistance and *ompC* (encoding the outer membrane porin) expression. Expression levels were assessed in 26 imipenem-resistant *M. morganii*, 15 imipenem-intermediate strains, 15 imipenem-susceptible strains, and the reference strain *M. morganii* ATCC 25830. qRT-PCR results showed that expression levels of *ompC* did not differ significantly among the resistant, intermediate, and susceptible groups compared with the reference strain (**Figure 2**).

### 3.6 PBP activator-encoding gene expressions

**Figure 3:**
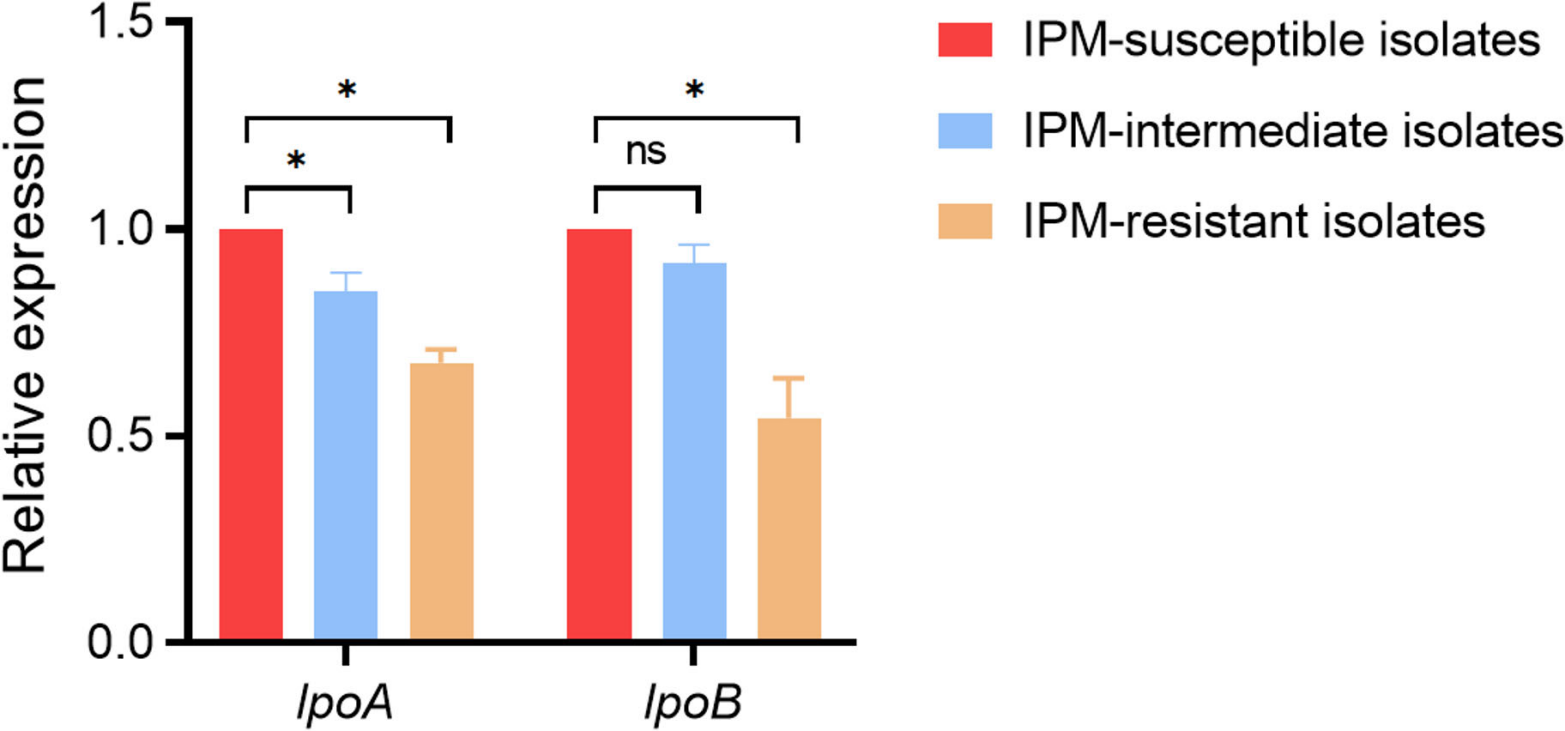
Relative expression level of PBP activator-encoding genes *lpoA* and *lpoB* among the 26 imipenem-resistant *M. morganii*, 15 imipenem-intermediate *M. morganii* strains, 15 imipenem-susceptible *M. morganii* groups. *P <0.05; n.s., *P* >0.05.

The expression of the PBP activator-encoding genes *lpoA* and *lpoB* was examined in 26 imipenem-resistant isolates, 15 imipenem-intermediate isolates, 15 imipenem-susceptible isolates, and the reference strain *M. morganii* ATCC25830. qRT-PCR analysis showed that, compared with the reference strain, *lpoA* and *lpoB* expression levels were reduced in the imipenem-resistant isolates. Significant differences in *lpoA* and *lpoB* expression were also observed among the resistant, intermediate, and susceptible groups (**Figure 3**).

## 4 Discussion

Infection by *M. morganii*, a rod-shaped Gram-negative bacterial pathogen, is characterized by its prevalence in nosocomial infections. First isolated in 1906 from the feces of a child, *M. morganii* belongs to the family Proteae, a subfamily of enteric bacteria (Liu et al., 2016; Laupland et al., 2022). The genome length of *M. morganii* is approximately 4 Mbp, encoding about 4,000 potential protein sequences. Historically, *M. morganii* was not considered clinically significant in relation to summer diarrhea, but since it has been detected in a variety of conditions, including urinary tract infections, it has been reclassified as an opportunistic pathogen capable of causing rare infections (Sakaguchi et al., 2014).

According to the latest analysis from the SENTRY antimicrobial resistance surveillance program, M. morganii ranks 12^th^ among Gram-negative organisms responsible for bloodstream infections (Zaric et al., 2021). Typically, *M. morganii* acquires resistance through mobile genetic elements; however, resistance may also result from gene alterations. There are many types of mobile genetic elements in bacteria, including plasmids, transposons, integrons, and insertion sequences. This mode of transfer increases the likelihood of acquiring antimicrobial resistance and leads to wider transmission of resistance in clinical settings (Bandy, 2020). In addition, resistance of *M. morganii* to fluoroquinolones, most aminoglycosides, and azithromycin increases the difficulty of anti-infective therapeutic management. Since carbapenems possess a broad spectrum of antimicrobial activity, they are extensively used in clinical settings to treat MDR Gram-negative pathogenic microorganisms (Timofte et al., 2016; Yao et al., 2025). Yet, several surveillance programs have reported that carbapenem resistance is increasing rapidly, making clinical treatment more challenging. Resistance of *M. morganii* to carbapenems is mainly attributed to the production of carbapenemases, including KPC-2 and New Delhi metallo-β-lactamase 1 (NDM-1) (Guo et al., 2019; Bonnin et al., 2024; Munir et al., 2025). It has been reported that *bla*_KPC-2_, located within the same mobile genetic element (an integrative structure consisting of a Tn3-based transposon and a partial fragment of Tn4401), may be transmitted between different plasmids in three *M. morganii* isolates by M Huang et al (Shen et al., 2009; Shi et al., 2012).

As a result, it is important to stress the need for monitoring carbapenem resistance in *M. morganii*. Moreover, clinical experience with treatment regimens for aggressive MDR or extensively drug-resistant (XDR) *M. morganii* infections should continue to be developed.

The type and content of PBPs vary among bacterial species, including high-molecular-weight enzymes PBP1a, PBP1b, PBP2, and PBP3, but PBPs of different bacteria share similar structures and functions. PBPs are the main targets of β-lactam antibiotics (Shalaby et al., 2020; Bertonha et al., 2023; Hu and Wang, 2024). These antibiotics specifically bind to PBPs on the inner membrane of bacterial cells, interfere with the normal enzymatic activity of PBPs, and consequently disrupt peptidoglycan synthesis. As a result, cell wall synthesis is blocked, ultimately leading to bacterial death (Lessel, 1996). Different β-lactam antibiotics bind to different PBPs, and changes in PBP structure or abundance are important mechanisms leading to bacterial resistance (Aissa et al., 2016; Cherkaoui et al., 2017). Even the same antibiotic can act on different PBPs depending on the bacterial species. For example, a study comparing the binding affinities of *Escherichia coli* PBPs with three antibiotics—methicillin, cephalexin, and cefradine—showed that methicillin bound specifically to PBP2, whereas cephalexin and cefradine had high affinity for PBP1a (Katayama et al., 2004). Doripenem also exhibits high affinity for PBPs in various species, such as PBP3 in *Pseudomonas*, PBP1, PBP2, and PBP4 in *Staphylococcus aureus*, and PBP2 in *E. coli*. Compared with imipenem, the stronger antipseudomonal activity of doripenem and meropenem against *Pseudomonas aeruginosa* may be attributed to their stronger binding affinity for PBP2 and PBP3 (Goffin and Ghuysen, 2002; Malik et al., 2020). It has also been reported that doripenem binds more efficiently to PBP1a and PBP1b, followed by PBP2, while imipenem is more likely to penetrate the bacterial cell wall and reach its target PBPs at a faster rate than other antibiotics in *Streptococcus pneumonia* (Cherkaoui et al., 2017). There is evidence that carbapenem resistance is caused by a reduction in expression or absence of the two main porins, combined with β-lactamase activity and PBP alterations (Breilh et al., 2013). However, PBP modification alone rarely leads to high levels of carbapenem resistance. In this study, we detected 26 imipenem-resistant *M. morganii* isolates; however, these strains remained susceptible to both meropenem and ertapenem and had low MIC values. In addition, the imipenem-resistant strains carried more specific PBP mutations than the imipenem-susceptible and imipenem-intermediate isolates, a finding consistent with previous studies.

It was reported many years ago that PBP enzymes ^Ec^PBP1a and ^Ec^PBP1b from *E. coli* may be specifically activated by the outer-membrane lipoproteins ^Ec^LpoA (activating ^Ec^PBP1a) and ^Ec^LpoB (activating ^Ec^PBP1b), respectively (Paradis-Bleau et al., 2010; Typas et al., 2010). Both activators interact directly with their cognate homologs to form a transenvelope complex (Egan et al., 2014; Jean et al., 2014; King et al., 2014). In *Gammaproteobacteria*, the expression level of LpoA was found to be relatively conserved (Typas et al., 2010). By contrast, LpoB and the UB2H regulatory domain of ^Ec^PBP1b to which it binds appeared to be largely restricted to the *Enterobacteriaceae*, despite the relatively broad distribution of PBP1b sequences (Typas et al., 2010). Imipenem exhibits uniquely high affinity for PBP1a and PBP1b in Enterobacteriaceae, relying critically on their activation by LpoA/LpoB for bactericidal activity. Downregulation of lpoA/lpoB impairs the transenvelope complex formation required for PBP1a/1b function, directly compromising imipenem binding. In contrast, meropenem and ertapenem primarily target PBP2/3 with minimal dependence on PBP1a/1b activation. This mechanistic distinction explains why lpoA/lpoB downregulation selectively confers imipenem resistance while preserving susceptibility to other carbapenems. In agreement with other studies (Dorr et al., 2014; Yin et al., 2015; Greene et al., 2018), we found that the expression levels of *lpoA* and *lpoB* in 26 imipenem-resistant *M. morganii* isolates were decreased compared with those in the reference strain *M. morganii* ATCC25830. In addition, significant differences in *lpoA* and *lpoB* expression were observed among the resistant, intermediate, and susceptible groups. These findings confirm that reduced expression of the PBP activator genes *lpoA* and *lpoB* may contribute to imipenem resistance in clinical *M. morganii* isolates.

## 5 Conclusion

In conclusion, this study is the first to investigate the clinical characteristics, specific resistance patterns, and molecular mechanisms of carbapenem-resistant *M. morganii* clinical isolates. These findings indicate that *M. morganii* clinical isolates showed distinct patterns and mechanisms of resistance to carbapenem antibiotics, which were different from those of other *Enterobacteriaceae*. *M. morganii* clinical isolates were commonly non-susceptible to imipenem, while imipenem-non-susceptible strains remained susceptible to meropenem and ertapenem. The low expression of the PBP activator-encoding genes *lpoA* and *lpoB* was the main underlying mechanism among the imipenem-resistant *M. morganii* isolates that remained susceptible to meropenem and ertapenem, along with the presence of specific PBP mutations. Meanwhile, the overexpression of efflux pump might also contribute to imipenem resistance in *M. morganii*. With carbapenems being increasingly used as therapeutic options, it is urgent to establish monitoring programs to prevent the spread of carbapenem resistance.

## Data Availability

The original contributions presented in the study are included in the article/**Supplementary Material**. Further inquiries can be directed to the corresponding author.
