# Supplementary material for: Clinical characteristics, specific resistance patterns, and molecular mechanisms of carbapenem-resistant Morganella morganii isolates
Source: Front Cell Infect Microbiol. 2025 Sep 5;15:1672736. doi: 10.3389/fcimb.2025.1672736 (PMC12446265; doi:10.3389/fcimb.2025.1672736)
Supplement: Supplementary file 1 [file Table1.docx]

**TABLE S1 Primers used in the study**

| Genes | Primer sequence（5’→3’） | Annealing temperature (℃) | Amplicon size (bp) |
| --- | --- | --- | --- |
| *bla*_KPC_ | F: GCTACACCTAGCTCCACCTTC  R: TCAGTGCTCTACAGAAAACC | 52 | 1050 |
| *bla*_NDM_ | F: GGTTTGGCGATCTGGTTTTC  R: CGGAATGGCTCACGATC | 52 | 621 |
| *bla*_IMP_ | F: CATGGTTTGGTGGTTCTTGT  R: ATAATTTGGCGGACTTTGGC | 50 | 488 |
| *bla*_VIM_ | F: GATGGTGTTTGGTCGCATA  R: CGAATGCGCAGCACCAG | 58 | 390 |
| *bla*_OXA-48_ | F: TTGGTGGCATCGATTATCGG  R: GAGCACTTCTTTTGTGATGGC | 58 | 744 |
| *bla*_CTX-M-1_ | F: AAAAATCACTGCGTCAGTTCAC  R: ACAAACCGTTGGTGACGATT | 55 | 867 |
| *bla*_CTX-M-9_ | F: TAT TGGGAGTTTGAGATGGT  R: TCCTTCAACTCA GCAAAAGT | 50 | 933 |
| *bla*_SHV_ | F: AGCCGCTTGAGCAAATTAAAC  R: ATCCCGCAGATAAATCACCAC | 60 | 713 |
| *bla*_TEM_ | F: CATTTCCGTGTCGCCCTTATTC  R: CGTTCATCCATAGTTGCCTGAC | 60 | 800 |
| *mrcA* ^a^ | F: CTGTTTTTATCCGCTTAATCAGAA  R: CGGAAATTTTCAGGTGAAGTT | 58 | 2526 |
| *pbpC* ^b^ | F: CGGTCAGAATGAGCGGCATCC  R: GTTTACAATTTACCTGTCCCT | 54 | 2361 |
| *mdrA* ^c^ | F: CACAAGCAACGGATGGACTTC  R: GGGCATCACTCTGTTTCAC | 55 | 1881 |
| *lpoA* | F: GTTAGCATCAGCCAGGTT  R: ACAGGTTCAGCAATATCAGA | 50 | 147 |
| *lpoB* | F: GGTGATAACGATACACTGAC  R: AGTTGCATCTCCATCTGAC | 50 | 125 |
| *ompC* | F: CCTGCTGACCTACCGTAA  R: CGTGCTGCGATAGAGTTAT | 50 | 102 |

^a^ PBP1a encoded by the *mrcA* gene; ^b^ PBP1c encoded by the *pbpC* gene; ^c^ PBP2 encoded by the *mdrA* gene.
